# Why orthopaedics and trauma surgery loses part of its future workforce – a cross-sectional study of gender differences in medical students’ specialty choice

**DOI:** 10.1186/s12909-025-08320-2

**Published:** 2025-11-22

**Authors:** Conrad Ketzer, Amelie Sinz, Gregor Bielesch, Carolin Müller, Andrea Achtnich, Peter Biberthaler, Marc Hanschen, Olivia Bohe

**Affiliations:** 1 Department of Trauma Surgery, TUM University Hospital, Klinikum rechts der Isar, Ismaninger Str. 22, 81675 Munich, Germany; 2 Department of Orthopaedics and Sports Orthopaedics, TUM University Hospital, Klinikum rechts der Isar, Ismaninger Str. 22, 81675 Munich, Germany

**Keywords:** Specialty choice, Gender disparities, Medical students, Orthopaedics, Trauma surgery, Career preference, Medical education

## Abstract

**Background:**

As patient volumes rise and the workforce ages, medical specialties face mounting pressure to attract and retain new talent. Despite women making up the majority of medical students in Germany, their representation in surgical fields, especially orthopaedics and trauma surgery (O&T), remains comparatively low and has shown little improvement over the past decade. To ensure the future of surgical care, it is essential to understand how medical students – particularly women – perceive O&T and what factors shape their specialty choices.

**Methods:**

In this prospective cross-sectional survey, 676 medical students from 32 medical faculties in Germany and Austria completed a questionnaire to assess specialty preferences, career motivations, educational experiences, and attitudes toward potential reforms in specialty training. Descriptive statistics, group comparisons, and regression analyses were used to examine gender differences and trends across stages of medical education.

**Results:**

O&T was selected as a preferred specialty by 11.5% of respondents, with no significant gender difference at baseline (*p* = 0.213). However, female interest declined significantly throughout medical school, while male interest remained stable (*p* = 0.028).

The most important influencing factors were subject-related interest, work environment, and practical clinical experiences. Female students rated work–life balance, part-time options, and gender representation significantly higher than male peers (all *p* < 0.01). Only 67.9% of students considered O&T a suitable field for women. More than half reported having been actively discouraged from entering the field—frequently by O&T physicians themselves (accounting for 30% of reported discouragement).

**Conclusion:**

This study reveals a persistent gap between early interest in O&T and the structural realities that may shape students’ long-term career decisions. This disconnect does not appear to stem from a lack of interest or ability but may be linked to perceived barriers such as rigid hierarchies, limited female representation, and exclusionary workplace cultures. Supporting future talent may therefore require attention to sustainable career paths, inclusive leadership, and visible efforts toward structural change.

**Supplementary Information:**

The online version contains supplementary material available at 10.1186/s12909-025-08320-2.

## Background

Current discussions about the future of orthopaedic and trauma surgery (O&T) no longer only revolve around technological innovation, but around who will be left to perform it [1–3]. The question of how to secure the next generation of physicians has become central to the specialty’s workforce debate [1–3].

The shortage of surgical professionals is well established in recent literature, with data consistently highlighting a growing mismatch between demand and surgeons [3–5]. Nearly 30% of outpatient physicians are over the age of 60, one-third of all contracted doctors work part-time, and nearly a quarter are retired or no longer engaged in direct patient care [2, 6–9]. By 2030, an estimated 7,300 surgical posts could remain vacant, while patient numbers continue to increase [4, 5, 10].

This development coincides with a shift in the medical student population. Although women now make up approximately 64% of all medical students in Germany, their representation in O&T has remained low and largely unchanged for years [11, 12]. This persistent mismatch reflects more than a lag in representation; it signals a deeper structural disconnect between early interest and long-term engagement [13].

The consequences are already visible in clinical practice. In a nationwide survey, 80% of chief and senior physicians reported that the current shortage of applicants is affecting care quality [1, 2].

This study examines how the current generation of medical students perceives O&T, particularly in terms of gender-related barriers. The aim is to identify key factors influencing specialty choice, examine gender-specific barriers, and assess how O&T can become more attractive to the next generation.

## Methods

This prospective cross-sectional study examined the determinants of specialty choice among medical students, with a particular focus on gender differences and the field of O&T. The survey targeted undergraduate medical students from all years of study at medical faculties in Germany and Austria. Recruitment was conducted through both analogue and digital channels, including posters in lecture halls and libraries, as well as targeted outreach via university-specific social media groups, faculty websites, and medical societies. Participation was entirely voluntary, anonymous, and without compensation. Ethical approval was obtained from the Ethics Committee of the Technical University of Munich (Reference No.: 2024-321-S-SB). A clinical trial number was not applicable.

Data collection was conducted between November 2024 and February 2025 using a self-developed questionnaire. The instrument was developed based on a comprehensive literature review on specialty choice and gender disparities [13–17]. It has not been published or used in previous studies. To ensure content validity, the questionnaire was reviewed by board-certified orthopaedic and trauma surgeons and pilot-tested with 15 medical students from different semesters at the University Hospital of the Technical University of Munich. Based on their feedback, minor adjustments were made to improve clarity and comprehensibility. An English version of the final questionnaire is provided as Supplementary 1.

The questionnaire was administered via EvaSys (V9.1, evasys GmbH, Munich), a secure online survey platform hosted by the Technical University of Munich. It consisted of 23 items, including Likert-scale and multiple-choice questions, as well as two open-ended items. Most items used five-point Likert scales, ranging from 1 (“not at all important”) to 5 (“very important”) or from 1 (“no influence at all”) to 5 (“very strong influence”). In addition to sociodemographic data (age, gender, year of study, university), the survey assessed students’ intended career paths, current specialty preferences, and the relevance of various influencing factors. It also addressed experiences with gender-based discrimination and included ratings of proposed measures to improve the attractiveness of O&T.

Data analysis was performed using IBM SPSS Statistics (version 29.0). Descriptive statistics included frequency distributions, means (M), and standard deviations (SD). Group comparisons (e.g., by gender or year of study) were conducted using t-tests, Mann-Whitney U tests, or chi-square tests, as appropriate. A p-value < 0.05 was considered statistically significant. Likert-scale data were summarized using means and medians. Open-ended responses were coded and analysed using inductive thematic content analysis to identify recurring themes and patterns. Binary logistic regression models were used to examine associations between key predictors (e.g., gender, semester, work–life balance, discrimination, mentoring, hierarchy) and binary outcomes such as interest in or preference for O&T. Results are reported as odds ratios (OR) with 95% confidence intervals (CI).

The survey was conducted in full compliance with European data protection regulations. No personally identifiable data were collected or stored, ensuring complete anonymity of all participants.

## Results

### Sample

A total of 676 medical students participated in the survey. Of these, 73.5% (*n* = 496) were female, 25.8% (*n* = 174) were male, and 0.7% (*n* = 5) identified as non-binary. The mean age was 24.3 years (SD 3.5, range 18–38), with 60.5% of participants aged between 21 and 25 years. Most respondents were in the clinical phase of their studies, with the largest group enrolled in their 5th to 10th semesters (mean = 7.4; range: 1st–15th semester). Due to the small number of non-binary respondents (*n* = 5), gender-specific analyses were limited to female and male students. Participant characteristics are summarized in Table 1.

Study participants were enrolled at 32 medical faculties in Germany and Austria. The four most frequently represented institutions were Ludwig-Maximilians-University Munich (*n* = 86), Technical University Munich (*n* = 65), Martin-Luther-University Halle-Wittenberg (*n* = 55), and Ruhr-Universität Bochum (*n* = 54).

**Table 1:** Demographic characteristics of the study sample

Characteristic	*n* = 676
**Age**	
Age, mean (SD)	24.3 (3.5)
Age, median (range)	24 (18–38)
**Sex**	
Female	496 (73.5%)
Male	174 (25.8%)
Non-binary	5 (0.7%)
**Semester**	
Semester, mean (SD)	7.36 (3.4)
Semester, median (range)	7 (1–15)

Data are presented as mean (SD), median (range), or n (%). “n” denotes the number of valid responses for each variable. Age (*n* = 659), sex (*n* = 675), semester (*n* = 675), refer to valid responses

### Specialty choice and career intentions

A large majority of respondents (94.7%, *n* = 640) indicated that they planned to pursue a medical career after graduation, while only 5.3% (*n* = 36) stated otherwise. At the time of the survey, 38.2% (*n* = 258) had already chosen a medical specialty, 61.8% (*n* = 418) had not yet made a final decision. The likelihood of having selected a specialty increased significantly with academic seniority (β = 0.194, *p* < 0.001), ranging from 27.0% in the 1 st semester to 82.6% in the 13th.

Among the most frequently selected specialties were anaesthesiology (11.8%, *n* = 79), O&T (11.5%, *n* = 77) and paediatrics (11.5%, *n* = 77). Other common preferences included obstetrics and gynaecology (7.6%, *n* = 51) and general practice (7.0%, *n* = 47).

Among male students, 14.5% (*n* = 25) selected orthopaedics and trauma surgery, compared to 10.5% (*n* = 52) of female students (*p* = 0.213). Additional data on career intentions and specialty preferences are presented in Table 2.

**Table 2:** Distribution of most selected specialty choices in our study cohort

Speciality	Percentage of Respondents (%)	Female (%)	Male (%)	*p*-value
Anaesthesiology	11.8	11.2	13.9	n.s.
Orthopaedics and Trauma Surgery	11.5	10.5	14.5	n.s.
Paediatrics	11.5	13.2	6.4	0.002
Obstetrics and Gynaecology	7.6	9.7	1.7	0.001
General Practice	7.0	7.5	4.6	n.s.
Internal Medicine	7.0	5.7	10.4	n.s.
Neurology	5.7	6.1	4.6	n.s.
General Surgery	4.2	4.3	4.0	n.s.
Cardiology	3.9	2.4	8.1	0.002
Psychiatry	3.3	3.0	3.5	n.s.

This table presents the percentage distribution of the ten most frequently reported specialty preferences among the medical students who answered this question (n = 672). Paediatrics (p < 0.05), Obstetrics and Gynaecology (*p* < 0.05), and Cardiology (*p* < 0.05) showed statistically significant differences between female and male respondents. All other differences were not statistically significant and are labelled as “n.s.” (not significant).

### Factors influencing specialty choice

Overall, the most important criterion in choosing a specialty was interest in the field (M = 4.80, SD = 0.45). This was followed by the work environment and culture (M = 4.44, SD = 0.79), compatibility with family life (M = 4.20, SD = 0.96), and work–life balance (M = 4.03, SD = 0.90). Other factors such as the possibility to enter private practice (M = 3.69, SD = 1.17), part-time work opportunities (M = 3.47, SD = 1.22), income (M = 3.36, SD = 0.97), and career opportunities (M = 3.33, SD = 1.03) were rated as moderately important.

When comparing genders, female students rated work–life balance (M = 4.12) significantly higher than male students (M = 3.75; *p* < 0.001), as well as compatibility with family life (M = 4.29 vs. 3.99; *p* = 0.001), part-time work opportunities (M = 3.72 vs. 2.77; *p* < 0.001), and the work environment and culture (M = 4.51 vs. 4.22; *p* < 0.001). In contrast, male students placed significantly more importance on income prospects (M = 3.61 vs. 3.28; *p* < 0.001) and career opportunities (M = 3.67 vs. 3.22; *p* < 0.001).

Female students rated the impact of night and shift work (M = 3.56 vs. 3.29; *p* = 0.005) and the gender distribution in the field (M = 2.53 vs. 2.00; *p* < 0.001) as more influential. Male students attributed greater importance to emergency situations (M = 3.66 vs. 3.43; *p* = 0.019), status and prestige (M = 2.51 vs. 2.03; *p* < 0.001), and academic opportunities (M = 2.81 vs. 2.53; *p* = 0.016). No significant gender differences were found in the perceived influence of manual or surgical activities (*p* = 0.938) or acceptance of workload and competition (*p* = 0.116). Detailed results on the importance of influencing factors and gender differences are presented in Table 3.

**Table 3:** Factors Influencing Speciality Choice of Medical students

Influencing factors	Mean	SD	Female	Male	*p* - value
Interest in the specialty	4.80	0.45	4.81	4.76	n.s.
Manual and surgical activities	3.32	1.26	3.32	3.33	n.s.
Acceptance of higher workload and competition	2.99	1.11	2.96	3.10	n.s.
Work environment and culture	4.44	0.79	4.51	4.22	0.001
Working hours and work-life balance	4.03	0.90	4.12	3.75	0.001
Possibility of part-time work	3.47	1.22	3.72	2.77	0.001
Role models and mentors within the specialty	3.65	1.12	3.70	3.49	0.048
Gender distribution within the specialty	2.40	1.23	2.53	2.00	0.001
Status and prestige of the specialty	2.15	1.11	2.03	2.51	0.001
Possibility to establish a private practice	3.69	1.17	3.73	3.61	n.s.
Income prospects	3.36	0.97	3.28	3.61	0.001
Quality of teaching	3.89	1.03	3.99	3.67	0.001
Academic work/doctoral research	2.82	1.20	2.86	2.73	0.015
Lectures	2.76	1.10	2.78	2.74	n.s.
Personal experiences during clerkships and final year	4.35	0.84	4.42	4.13	0.001

Table 3 presents the mean (M) and standard deviation (SD) of the perceived importance of various factors influencing specialty choice among medical students (n=673). The Likert scales ranged from 1 (“not at all important” / “no influence at all”) to 5 (“very important” / “very strong influence”). Significant gender differences (*p* < 0.05) are indicated with the corresponding p-value; non-significant results are labelled as “n.s.” (not significant)

### Educational experiences

Overall, 41.2% of students reported prior clinical exposure to O&T (clerkship or rotation), whereas 58.7% had none; exposure did not differ by gender (*p* = 0.591). Educational experiences played a significant role in shaping specialty preferences. Clinical placements during clerkships and the final clinical year were rated as most influential (M = 4.35, SD = 0.84), followed by the quality of teaching (M = 3.89, SD = 1.03), hands-on training sessions (M = 3.69, SD = 1.11), and the influence of mentors and role models within a specialty (M = 3.65, SD = 1.12). In contrast, lectures (M = 2.76, SD = 1.10) and scientific work, such as doctoral research (M = 2.82, SD = 1.20), were perceived as less influential overall.

Female students placed greater importance on clerkships and the final year (M = 4.42 vs. 4.13; *p* = 0.001), teaching quality (M = 3.99 vs. 3.67; *p* = 0.001), hands-on training (M = 3.76 vs. 3.50; *p* = 0.012), and mentorship (M = 3.70 vs. 3.49; *p* = 0.048). A detailed comparison of educational influences by gender is presented in Table 3.

### Interest in O&T over time

When asked about their general interest in pursuing a career in O&T, 20.8% expressed high or very high interest, 59.5% reported low or very low interest, and 19.7% indicated moderate or neutral interest. The gender-specific analysis revealed no significant difference in overall interest levels (female: M = 2.35 vs. male: M = 2.51; *p* = 0.156). However, a divergent trend emerged over the course of medical training: among female students, interest in O&T significantly declined with increasing semester level (β = − 0.036; *p* = 0.028), while interest among male students remained stable (β = − 0.001; *p* = 0.981).

A logistic regression model identified general interest in O&T as the strongest positive predictor of choosing the field (β = 2.87; *p* < 0.001), with a significantly higher likelihood (β = 1.07; *p* = 0.019). Other factors such as gender, semester, work–life priorities, or previous experiences of discrimination showed no significant independent effects.

### Barriers to choosing O&T

To assess the barriers that discourage medical students from pursuing a career in O&T, respondents were asked to indicate which factors deterred them. The most frequently cited reason was a lack of personal interest (57.8%, *n* = 391), followed by concerns about hierarchical structures (50.3%, *n* = 340) and difficulties reconciling career and family life (46.6%, *n* = 315). Additional common concerns included high stress levels (41.6%, *n* = 281) and workload (36.4%, *n* = 246), physically demanding work (30.8%, *n* = 208), and perceptions of gender-related disadvantages (34.8%, *n* = 235).

**Fig. 1:**
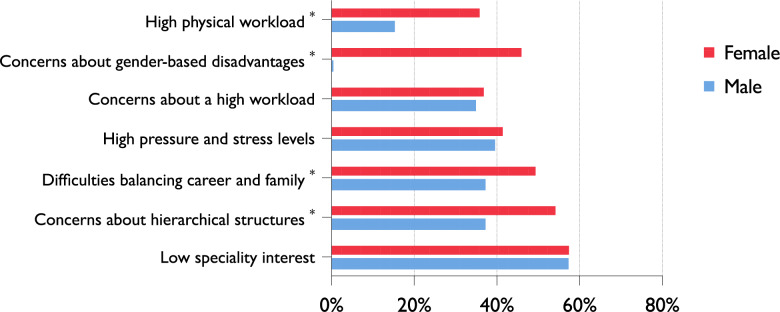
Gender Differences in Deterring Factors from Pursuing O&T (*n* = 676). Reported reasons for not choosing O&T as a specialty, stratified by gender. Female students were significantly more likely to cite concerns about gender-related disadvantages, hierarchical structures, difficulties balancing career and family, and high physical workload (all *p* < 0.01). * indicates factors with significant differences

Gender-specific analyses revealed striking differences. Female students were significantly more likely than their male counterparts to cite gender-based disadvantages (46.4% vs. 0.6%; *p* < 0.001), concerns about physical demands (36.3% vs. 15.5%; *p* < 0.001), and difficulties balancing family and career (49.8% vs. 37.4%; *p* = 0.006). Similarly, worries about hierarchical structures were more prevalent among female students (54.6% vs. 37.4%; *p* < 0.001). In contrast, no significant gender differences emerged in reporting high stress levels (41.9% vs. 39.7%; *p* = 0.663) or concerns about workload (37.3% vs. 35.1%; *p* = 0.663). An overview of the most frequently reported barriers to choosing O&T is provided in Fig. 1.

### Strategies to improve the attractiveness of O&T

To assess how the attractiveness of O&T could be improved, students were asked to evaluate a range of proposed measures. The highest support was given to improving the work environment and team culture (M = 4.27; SD = 1.00), followed by enabling family–career compatibility (M = 4.06; SD = 1.10) and implementing flexible working hours and reducing overtime (M = 3.98; SD = 1.11). Structural reforms were also widely endorsed, including flattening hierarchies (M = 4.04; SD = 1.13) and launching awareness campaigns to address stereotypes and bias (M = 3.82; SD = 1.25).

Gender-specific analyses revealed notable differences. Female students consistently rated nearly all measures significantly higher than male students, including work environment (M = 4.34 vs. 4.06; *p* = 0.003), family-friendly models (M = 4.14 vs. 3.85; *p* = 0.003), flexible structures (M = 4.05 vs. 3.80; *p* = 0.013), flattening hierarchies (M = 4.11 vs. 3.83; *p* = 0.008), and addressing bias and stereotypes (M = 3.98 vs. 3.36; *p* < 0.001).

**Fig. 2:**
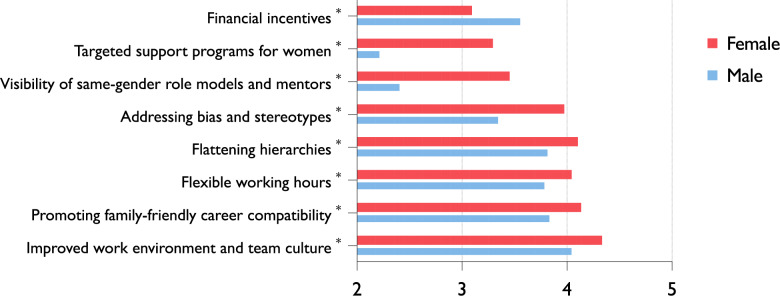
Gender Differences in Support for Strategies to Improve the Attractiveness of O&T. Students rated various proposed measures on a 5-point Likert scale. Female students consistently rated nearly all strategies significantly higher than male students (*p* < 0.05), particularly improving the work environment, enabling family–career compatibility, flexible working hours, flattening hierarchies, promoting same-gender mentors, and targeted support for women. Male students showed higher endorsement for financial incentives (*n* = 676). * indicates factors with significant differences

Further differences emerged regarding the role of representation and gender-specific support structures. Female students placed significantly more value on increasing the visibility of same-gender role models and mentors within the field (M = 3.70 vs. 3.49; *p* = 0.0048), as well as on targeted support and career development programs for women (M = 3.30 vs. 2.23; *p* < 0.001). Male students, in contrast, assigned greater importance to financial incentives (M = 3.57 vs. 3.10; *p* < 0.001). Figure 2 summarizes the levels of support for proposed strategies to improve the attractiveness of O&T, stratified by gender.

The qualitative analysis of the open-ended responses revealed a recurring pattern of concerns, including toxic or unsupportive work environments, limited practical training opportunities, and experiences of gender discrimination. Additional barriers mentioned were the physically demanding nature of the specialty, long working hours, poor work-life balance, and a lack of intellectual variety.

### Gender-Based barriers and discriminatory experiences

**Fig. 3:**
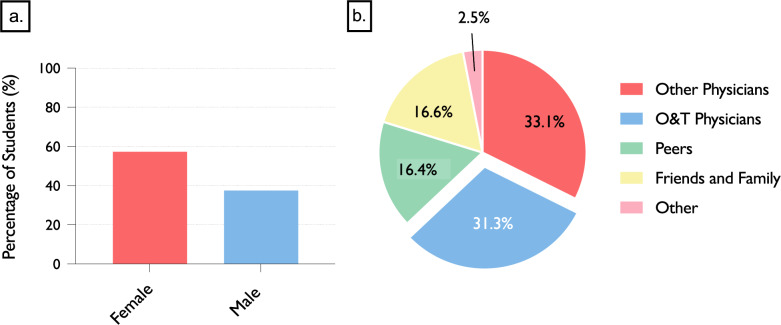
Sources and Gender Differences in Discouragement from Pursuing O&T. (**a**) Proportion of students who reported having been actively discouraged from pursuing a career in O&T, stratified by gender. Female students reported significantly higher rates of discouragement (57.3%) compared to male students (37.6%) (*n* = 672, *p* < 0.001). (**b**) Sources of discouragement as reported by affected students. A total of 718 individual instances were recorded. Most frequently, discouragement came from physicians of other specialties (33.1%) or O&T physicians themselves (31.3%), followed by friends or family (16.6%), peers (16.4%), and other sources (2.5%)

A total of 64.5% of female students (*n* = 316) and 76.7% of male students (*n* = 132) considered O&T an appropriate specialty for women (*p* = 0.005). More than half of the female students (57.3%, *n* = 283) reported being actively discouraged from pursuing O&T, compared to 37.6% of male students (*n* = 67, *p* < 0.001). Among those who reported such discouragement (*n* = 352), a total of 718 sources were identified. These included physicians from other specialties (33.1%, *n* = 238), orthopaedic and trauma surgeons themselves (31.3%, *n* = 225), friends and family (16.6%, *n* = 119), fellow students (16.4%, *n* = 118), and others (2.5%, *n* = 18). Figure 3 illustrates the gender distribution and sources of active discouragement from choosing O&T.

For many students, gender played a tangible role in their specialty choice. Overall, 53.0% (*n* = 357) stated that their gender had influenced their decision-making. This proportion was significantly higher among female students (62.4%, *n* = 309) than among male students (24.9%, *n* = 43; *p* < 0.001).

Almost half of all participants (47.0%, *n* = 317) reported having experienced gender-based discrimination during their studies or clinical training. Again, female (56.3%, *n* = 278) were affected much more frequently than their male peers (20.1%, *n* = 35; *p* < 0.001).

Specifically, within O&T, 18.2% (*n* = 122) of students had encountered negative gender-related experiences. Here again, women were disproportionately affected (24.2%, *n* = 119), whereas such reports were virtually absent among male students (0.6%, *n* = 1; *p* < 0.001).

**Fig. 4:**
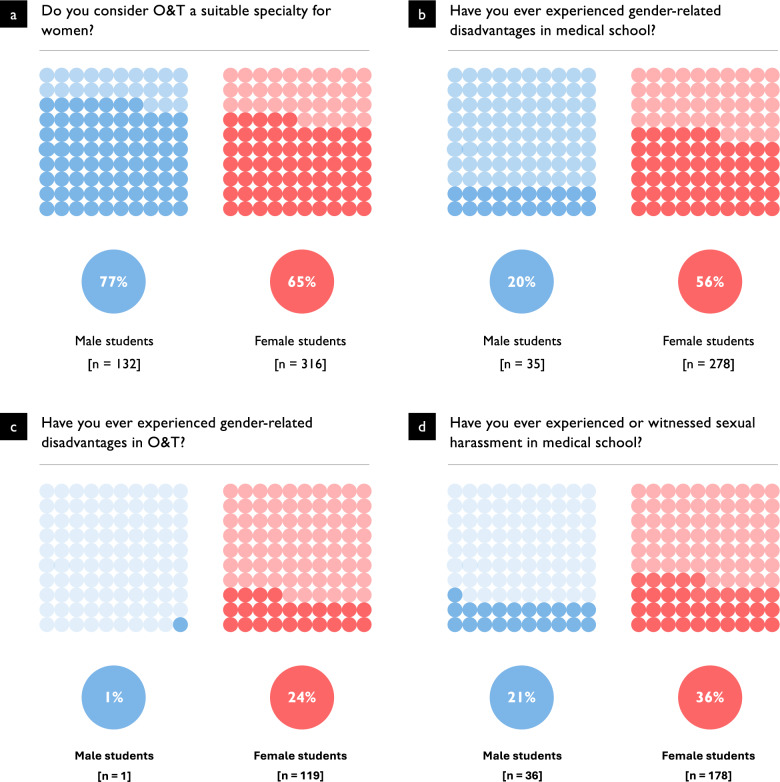
Gender-Based Perceptions and Experiences in O&T. Numbers in brackets (n) show how many students answered ‘yes’ to the item. (**a**) Perceived suitability of O&T as a specialty for women, stratified by gender. Female students were significantly less likely than male students to consider O&T suitable for women (64.5% vs. 76.7%; p = 0.0042; n = 667). (**b**) Reported experiences of gender-related disadvantages during medical education. Female students were significantly more likely than male students to report such experiences (56.3% vs. 20.1%; *p* < 0.0001; n = 674). (**c**) Gender-related disadvantages within O&T among students with prior exposure to the field. Female students were significantly more likely than male students to report negative experiences (24.2% vs. 1.0%; *p* < 0.0001; n = 672). (**d**) Sexual harassment (experienced or witnessed) during medical education. Female students were significantly more likely than male students to report such experiences (36.0% vs. 21.0%; *p* < 0.0001; n = 676)

The study also addressed the issue of sexual harassment during medical education. In total, 32.2% of students (*n* = 218, 81.7% of these were female) reported that they had either witnessed or personally experienced sexual harassment. An additional 16.0% (*n* = 108) expressed uncertainty in this regard. Figure 4 summarizes gender-related perceptions and experiences in O&T, highlighting significant disparities.

Open-ended responses (*n* = 110) consistently highlighted recurring themes of gender-based discrimination. Female students reported a range of experiences, including derogatory comments that women are “too emotional” or “too weak” for surgical specialties, as well as suggestions to consider other fields perceived as more suitable for women. Many accounts described systematic exclusion from practical training opportunities and the preferential treatment of male colleagues in surgical settings. Occasional incidents of sexual harassment and inappropriate remarks were also mentioned. These themes emerged as the most frequently reported issues in the open-ended responses.

## Discussion

The growing demand for physicians in Germany contrasts sharply with a rising trend of licensed doctors stepping away from direct patient care, alongside a doubling of part-time employment over recent years [2, 6, 7]. While this phenomenon is frequently attributed to generational shifts in work attitudes, our findings challenge this narrative: 94.7% (*n* = 640) of surveyed students expressed a clear intention to pursue a medical career regardless of gender, semester, or prior clinical experience. Consequently, attrition in later career stages appears to be less linked to motivational deficits and may instead reflect structural working conditions, echoing previous findings on hierarchies, limited autonomy, and exclusionary workplace cultures [2, 14, 18–20].

This study aimed to evaluate the main factors influencing current medical students in their choice of specialty with a focus on gender-specific differences and the field of O&T.

Overall, the strongest determinants were interest in the specialty itself, followed closely by the work environment and culture, practical experiences during clerkships, compatibility with family life and work–life balance. Our results resonate with broader evidence from both international reviews and national surveys, which identified these factors as key drivers of specialty choice [17, 21].

When examining gender-specific preferences, female students in our cohort placed greater emphasis on predictable working hours, flat hierarchies, and supportive mentoring structures. Male students, by contrast, more frequently prioritized financial rewards, career opportunities, research, and prestige. These differences mirror broader evidence showing that women tend to weigh compatibility with family life, structural flexibility, and supportive cultures more strongly, whereas men more often highlight prestige and career advancement [14, 15, 17].

O&T was among the most frequently selected specialties in this study, with no statistically significant gender difference observed overall (14.5% male vs. 10.6% female students; *p* = 0.213). However, while male interest remained stable across the duration of medical training, female interest declined significantly (β = – 0.036; *p* = 0.028). This trend reflects the current workforce composition in O&T, in which only 18.6% of board-certified specialists in Germany are women, and a mere 5,1% of female O&T surgeons hold leadership positions [8, 12, 16].

The decline in female interest in O&T over the course of medical school is often attributed to the perceived intensity of surgical work characterized by long hours, physical strain, and a high procedural load [15]. However, our data do not support this assumption: we found no significant gender differences in the acceptance of workload or surgical activities. This observation is in line with prior research noting that gynaecology—also a high-intensity, procedure-driven specialty—continues to attract a high proportion of women in German-speaking countries [22].

Furthermore, prior studies suggest that work characteristics alone are insufficient to explain specialty segregation. Riedel et al. reported that women in gynaecology experience fewer exclusionary dynamics, more accessible mentoring, and stronger symbolic belonging compared to other surgical fields [17]. Similarly, Diderichsen et al. demonstrated that gender segregation across specialties cannot be explained by workload alone but is closely linked to cultural perceptions and symbolic boundaries within medicine [14]. Our results are consistent with this view: while female interest in O&T declines, it remains comparatively stable in other specialties with similar workload demands, such as gynaecology, where women report more inclusive environments and stronger mentoring structures [22–24].

If neither workload nor the surgical content explains the decline in female interest in O&T, other factors must be explored. Although access to clinical placements was nearly equal across genders (41.2%; *p* = 0.591), their impact differed significantly. Compared to gynaecology, where 78% of male students report insufficient hands-on experience according to recent studies, O&T appears formally accessible for both genders [17]. Nonetheless, 58.7% of students in our cohort had not yet gained any practical exposure to O&T, highlighting a considerable early gap in specialty-specific experience. Prior studies have shown that a lack of early exposure, often coupled with limited access to role models, represents a major barrier for medical students [23, 25]. Bernstein et al. found that required musculoskeletal instruction was associated with higher orthopaedic application rates overall (+ 12%); among women, the application rate was 2.0% vs. 1.1% in schools without such instruction (≈ 75% relative difference) [25]. This structural gap is partly explained by the medical curriculum: while Orthopaedics is listed as a distinct subject in the clinical phase, there is no compulsory O&T rotation. In the final clinical year, only a surgery tertial is mandatory, with O&T covered under general surgery. As a result, practical exposure to O&T is not guaranteed and students can complete their studies without hands-on contact with the field—an issue that similarly affects smaller surgical subspecialties even more [26].

In our study, practical experience during internships and the final clinical year were rated as one of the most decisive factors in specialty choice (M = 4.35). These experiences were particularly formative for female students, who attributed greater importance to both clinical training (M = 4.42 vs. 4.13; *p* = 0.001) and teaching quality (M = 3.99 vs. 3.67; *p* < 0.001). However, during these critical training periods, female students often appear discouraged. Open-ended responses revealed persistent patterns of exclusion, marginalization, and gender-based stereotyping. Some students reported being addressed with condescending nicknames, warned they were “too emotional and too weak” for surgery, or explicitly told that “women don’t belong in the operation room.” Others described being denied meaningful tasks during rotations or being advised to pursue “more suitable” specialties.

Additionally, more than half of all students (53.0%) reported having been told not to pursue O&T at some point during their training. Among female students, this rate was notably higher at 57.3%, compared to 37.6% of male students. In approximately one third of cases (33.1%), the discouragement came from physicians in other specialties, while in 31.3% it was voiced by O&T physicians themselves.

Furthermore, we examined the prevalence of sexual harassment during medical education. A total of 32% of respondents reported to have either witnessed or been victim to sexual harassment with women disproportionately affected. Although our survey did not specify the context, previous studies have demonstrated that harassment in clinical settings often occurs within strong hierarchical structures [27]. Jenner et al. identified strong departmental hierarchies as the only structural factor significantly associated with harassment in both male and female trainees, while Jagsi et al. demonstrated that such experiences not only remain widespread in academic medicine but also erode professional confidence and limit career advancement [27, 28].

This pattern is reflected in students’ assessments of the field. Only 64.5% of female students considered O&T a suitable field for women, compared to 76.7% of male students (*p* = 0.005). 62% of women reported that gender influenced their specialty choice—more than twice the proportion of men. The concept of “learned exclusion”—when repeated incongruence between students’ expectations and clinical culture leads to strategic withdrawal—may help explain this disparity. Diderichsen et al. similarly highlighted that structural barriers, masculine homosociality, and a lack of role models contribute to gender segregation in specialty choice, even when initial interests are comparable across genders [14].

In addition, this study evaluated potential strategies to enhance the attractiveness of O&T as a specialty. Improved workplace culture (M = 4.27), flexible work models (M = 3.98), and family–career compatibility (M = 4.06) were the most endorsed measures. Female students placed greater emphasis on female role models (M = 3.46 vs. 2.42) and gender-specific mentoring (M = 3.30 vs. 2.23). Structural reforms—flattened hierarchies (M = 4.04) and addressing bias (M = 3.82) were widely supported.

The consistency of these demands could point to a deeper mismatch between what students value and what the specialty signals. Although part-time models and flexible arrangements are in place, prior studies indicate that they are rarely supported by leadership or embedded in clinical routines—highlighting that flexibility on paper does not necessarily translate into flexibility in practice [29–32].

International comparisons demonstrate that structural reforms are not only feasible but effective. In Sweden and other Scandinavian countries, gender disparities in surgical specialty choice have largely vanished within systems characterized by flatter hierarchies, supportive work–life policies, and visible equity initiatives [15]. These examples suggest that culture, representation, and working conditions are not peripheral aspects but have been shown to influence who enters, remains in, or leaves a specialty [2, 14, 17].

This study has several limitations. First, as a cross-sectional online survey, it is inherently subject to selection and response biases. Although recruitment efforts targeted a broad range of universities, geographical clustering may limit the generalizability of our findings. Variations in institutional culture and curricular structure across sites could have influenced both student experiences and their perceptions of O&T. Nevertheless, the large sample size helps to mitigate some of these biases.

Second, a substantial proportion of participants had prior exposure to O&T, either through clerkships or personal interest. Such exposure may have biased responses in both directions: while it can foster genuine enthusiasm, prior studies have shown that negative experiences during clinical rotations may also result in disillusionment. Because our study did not stratify by quality or setting of exposure, the effect of this factor remains uncertain.

Third, all data were self-reported. Perceptions of discrimination, hierarchy, or mentorship quality are shaped not only by objective conditions but also by individual expectations and situational contexts. In addition, socially desirable responding cannot be ruled out, particularly for sensitive items concerning gender bias and harassment.

Finally, the study design precludes causal inference. We cannot determine whether reported barriers directly translate into career decisions, or whether students with pre-existing specialty preferences were more likely to perceive certain structures as problematic. Longitudinal studies will therefore be necessary to clarify the dynamic relationship between early perceptions, clinical experiences, and eventual specialty choice.

## Conclusion

This study highlights a persistent disconnect between early interest in O&T and the professional realities that shape students’ specialty choices. While the field retains broad appeal, many—particularly women—disengage during medical school. Our findings point to structural and cultural barriers, including rigid hierarchies, limited representation, and gender-specific disadvantages, as key influences on this trajectory. Addressing these perceived barriers—especially by improving workplace culture, mentoring opportunities, and work–life compatibility—may be critical for sustaining student interest in O&T and securing its future workforce.

## Supplementary Information

Supplementary Material 1.

## Data Availability

The datasets generated and analysed during the current study are not publicly available due to the sensitive nature of the data and the potential risk of indirect participant identification, but they are available from the corresponding author on reasonable request.

## Abbreviations

CI: Confidence Interval
M: Mean
n.s.: Not significant
O&T: Orthopaedic and Trauma Surgery
OR: Odds Ratio
SD: Standard Deviation
SPSS: Statistical Package for the Social Sciences
