# Supplementary material for: Why orthopaedics and trauma surgery loses part of its future workforce – a cross-sectional study of gender differences in medical students’ specialty choice
Source: BMC Med Educ. 2025 Nov 22;25:1675. doi: 10.1186/s12909-025-08320-2 (PMC12676830; doi:10.1186/s12909-025-08320-2)
Supplement: Supplementary file 1 — Supplementary Material 1. [file 12909_2025_8320_MOESM1_ESM.pdf]

Questionnaire: Choice of Medical Speciality

Influencing Factors and Gender-Specific Differences in Orthopaedics and Trauma Surgery (English Version)

1. Personal Information

1.1 How old are you?

____

1.2 What is your gender?

[ ] Female
[ ] Non-binary
[ ] Male
[ ] Prefer not to say

1.3 At which university do you study?

____

1.4 What semester are you currently enrolled in?

[ ] 1.	[ ] 4.	[ ] 7.	[ ] 10.	[ ] 13
[ ] 2.	[ ] 5.	[ ] 8.	[ ] 11.	[ ] 14
[ ] 3.	[ ] 6.	[ ] 9.	[ ] 12.	[ ] >14

1.5 Do you plan to work as a physician after graduation?

[ ] Yes
[ ] No
[ ] I'm not sure yet

1.6 Have you already chosen a speciality?

[ ] Yes
[ ] No
[ ] I'm not sure yet

1.7 Which of the following specialities do you currently prefer?

[ ] General Medicine
[ ] Anaesthesiology
[ ] Occupational Medicine
[ ] Ophthalmology
[ ] General Surgery
[ ] Dermatology and Venereology
[ ] Endocrinology
[ ] Gynaecology and Obstetrics
[ ] Gastroenterology
[ ] Otorhinolaryngology
[ ] Hygiene and Environmental Medicine
[ ] Internal Medicine
[ ] Cardiology
[ ] Paediatric Medicine
[ ] Paediatric Surgery
[ ] Child and Adolescent Psychiatry and Psychotherapy
[ ] Haematology and Oncology
[ ] Neurosurgery
[ ] Neurology
[ ] Nephrology
[ ] Nuclear Medicine
[ ] Orthopaedics and Trauma Surgery
[ ] Pathology
[ ] Pharmacology and Toxicology
[ ] Psychiatry
[ ] Psychosomatic Medicine &
[ ] Radiology
[ ] Physical and Rehabilitation Psychotherapy Medicine
[ ] Forensic Medicine
[ ] Urology
[ ] Other Speciality

2. Which factors are most important to you when choosing your speciality?

(1) not important at all, (2) not important, (3) neutral, (4) important, (5) very important

2.1 Interest in the speciality	(1)	(2)	(3)	(4)	(5)
2.2 Working hours and work-life balance	(1)	(2)	(3)	(4)	(5)
2.3 Earning potential	(1)	(2)	(3)	(4)	(5)
2.4 Career and advancement opportunities	(1)	(2)	(3)	(4)	(5)
2.5 Possibility of private practice	(1)	(2)	(3)	(4)	(5)
2.6 Possibility of part-time work	(1)	(2)	(3)	(4)	(5)
2.7 Work environment and culture	(1)	(2)	(3)	(4)	(5)
2.8 Compatibility of work and family life	(1)	(2)	(3)	(4)	(5)

3. How much influence do the following aspects have on your choice of speciality?

(1) no influence at all, (2) low influence, (3) moderate influence, (4) strong influence, (5) very strong influence

3.1 Manual work and surgery	(1)	(2)	(3)	(4)	(5)
3.2 Emergency situations	(1)	(2)	(3)	(4)	(5)
3.3 Night and shiftwork	(1)	(2)	(3)	(4)	(5)
3.4 Scientific work / research opportunities	(1)	(2)	(3)	(4)	(5)
3.5 Acceptance of higher workload and career competition	(1)	(2)	(3)	(4)	(5)
3.6 Status and prestige of the speciality	(1)	(2)	(3)	(4)	(5)
3.7 Gender distribution among colleagues	(1)	(2)	(3)	(4)	(5)

4. During your studies, which factors have been most important for your future choice of speciality?

(1) not important at all, (2) not important, (3) neutral, (4) important, (5) very important

4.1 Role models and mentors in the speciality	(1)	(2)	(3)	(4)	(5)
4.2 Personal experiences during internships and practical year	(1)	(2)	(3)	(4)	(5)
4.3 Quality of teaching	(1)	(2)	(3)	(4)	(5)
4.4 Practical exercises during the studies	(1)	(2)	(3)	(4)	(5)
4.5 Lectures	(1)	(2)	(3)	(4)	(5)
4.6 Student assistant jobs	(1)	(2)	(3)	(4)	(5)
4.7 Scientific work / doctoral thesis	(1)	(2)	(3)	(4)	(5)

5.

5.1 Have you already completed an internship or clinical rotation in orthopaedics and trauma surgery?

[ ] Yes [ ] No

5.2 How interested are you in pursuing a career in orthopaedics and trauma surgery?

[ ] Very low [ ] Low [ ] Medium [ ] High [ ] Very high

5.3 Which factors discourage you from pursuing a speciality in orthopaedics and trauma surgery? *(Multiple answers possible)*

[ ] Lack of interest in the speciality
[ ] Concerns about experiencing disadvantages due to gender inequality
[ ] Concerns about hierarchical structures
[ ] Concerns about physically demanding work
[ ] High pressure and stress levels
[ ] Difficulties in reconciling career and family life
[ ] Heavy workload
[ ] Other factors

5.4 If you selected 'Other factors', please specify:

____

6. Which improvements would make the speciality of orthopaedics and trauma surgery more attractive to you?

(1) not important at all, (2) not important, (3) neutral, (4) important, (5) very important

6.1 Improved work-life balance through flexible working hours and less overtime	(1)	(2)	(3)	(4)	(5)
6.2 Increase in the number of same-gender role models and mentors in the speciality	(1)	(2)	(3)	(4)	(5)
6.3 Availability of special support programmes for women	(1)	(2)	(3)	(4)	(5)
6.4 Reduction of physical strain through technological aids and teamwork	(1)	(2)	(3)	(4)	(5)
6.5 Improved work environment and atmosphere	(1)	(2)	(3)	(4)	(5)
6.6 Promotion of work-family compatibility through measures such as childcare	(1)	(2)	(3)	(4)	(5)
6.7 Increased financial incentives and compensation	(1)	(2)	(3)	(4)	(5)

6.8 Better access to resources and support during training	(1)	(2)	(3)	(4)	(5)
6.9 Reduction of hierarchical structures in favour of flatter hierarchies	(1)	(2)	(3)	(4)	(5)
6.10 Raising awareness and combating prejudice and stereotypes in the speciality	(1)	(2)	(3)	(4)	(5)

7.

7.1 Do you think orthopaedics and trauma surgery is a suitable speciality for women?

[ ] Yes [ ] No

7.2 Have you ever been advised against specialising in orthopaedics and trauma surgery?

[ ] Yes [ ] No

7.3 If so, who advised you against specialising in orthopaedics and trauma surgery?

[ ] Fellow students [ ] Friends and family

[ ] Orthopaedics and trauma surgeons [ ] Physicians from other specialities

[ ] Others

7.4 Do you feel that your gender influences your choice of speciality?

[ ] Yes [ ] No

7.5 Have you ever experienced unequal treatment during your studies or clinical training because of your gender?

[ ] Yes [ ] No

7.6 Have you ever had negative experiences in the field of orthopaedics and trauma surgery because of your gender?

[ ] Yes [ ] No

7.7 If yes, please describe:

____

____

____

____

7.8 Have you ever witnessed or experienced sexual harassment during your studies or clinical placements?

[ ] Yes [ ] No [ ] I’m not sure
